# Editorial: Advancements and challenges in Mpox research

**DOI:** 10.3389/fmicb.2026.1791735

**Published:** 2026-02-04

**Authors:** Qi Peng, Lingbao Kong

**Affiliations:** 1 Institute of Pathogenic Microorganisms, Jiangxi Agricultural University, Nanchang, China; 2 College of Bioscience and Engineering, Jiangxi Agricultural University, Nanchang, China; 3 Nanchang Key Laboratory of Animal Virus and Genetic Engineering, Jiangxi Agricultural University, Nanchang, China

**Keywords:** antiviral drug, clinical characteristics, disease model, monkeypox virus, monoclonal antibody

Mpox (historically known as monkeypox) is a zoonotic viral infectious disease caused by the monkeypox virus (MPXV), which belongs to the *Orthopoxvirus* genus of the Poxviridae family. MPXV was first identified in laboratory monkeys in 1958, and the first case in humans was reported in the Democratic Republic of the Congo in 1970. Since then, Mpox has predominantly been endemic in Central and West Africa. Mpox has gradually garnered global attention since 2022. Mpox was designated as a Public Health Emergency of International Concern (PHEIC) by the World Health Organization (WHO). MPXV is an enveloped double-stranded DNA virus with a genome of approximately 190 kb, encoding 190 proteins. The core gene is highly conserved and responsible for viral replication, transcription, and synthesis of viral structural proteins. Phylogenetic analysis revealed that MPXV can be divided into two clades: the Congo Basin clade and the West African clade, with the Congo Basin strain being highly pathogenic. This Research Topic compiles updated information on MPXV pathogenesis, diagnostic methods, and antiviral studies, which could be applied to control the spread of Mpox and prevent future epidemics.

The instant detection of infectious diseases is necessary to control the spread of epidemics and prevent the outbreak of newly emerging viruses. Laboratory diagnostic methods include nucleic acid detection, cell culture, electron microscopy observation, and antigen detection. However, these methods often have limitations, such as cross-reactivity and low accessibility. Xiangjun et al. developed monoclonal antibodies that target the Mpox A29 protein and established two high-performance diagnostic assays: a chemiluminescence enzyme immunoassay (CLEIA) and a colloidal gold immunochromatographic assay. The CLEIA method achieved a detection limit of 40 pg/mL with exceptional specificity, distinguishing MPXV from closely related orthopoxviruses, such as the cowpox and vaccinia viruses. The colloidal gold immunochromatographic assay offered rapid, instrument-free results with a detection limit of 0.25 ng/mL.

Animal models that rely on non-human primates (NHPs) and rodents are limited by ethical concerns, high costs, and biosafety requirements. Developing versatile alternative models for MPXV infection could accelerate our understanding of Mpox pathogenesis and drug discovery. Mudjahid et al. proposed that Drosophila melanogaster is a cost-effective and ethically accessible alternative model organism mainly due to its conserved innate immune signaling pathways and fundamental cellular processes that are targeted by viruses. This model enables the high-throughput screening of antiviral compounds and the functional analysis of viral proteins, complementing mammalian studies while reducing reliance on vertebrate models.

While repurposed drugs such as tecovirimat remain frontline therapies, recent research has identified novel natural compounds with broad anti-orthopoxvirus activity. Because of the genetic conservation between the vaccinia virus and the MPXV virus, Zhang et al. used the vaccinia virus as a model to investigate the antiviral effects of piceatannol, a polyphenol abundant in edible fruits and vegetables, *in vitro*. The authors found that piceatannol inhibits vaccinia virus at multiple lifecycle stages, including viral entry, replication, production of extracellular enveloped viruses (EEVs), and vaccinia virus dissemination. Piceatannol represents a promising candidate for broad *orthopoxvirus* treatment, highlighting the value of exploring natural products for antiviral development.

Clinically, studies focusing on high-risk populations have deepened our understanding of severe Mpox manifestations. Wang et al. conducted a multicenter retrospective analysis of Mpox pneumonia in HIV-infected patients, finding that Mpox pneumonia occurred almost exclusively in patients with CD4^+^ T-cell counts < 200/μL. Their study characterized distinctive radiological features and emphasized that consistent antiretroviral therapy (ART) significantly improved survival, underscoring the importance of integrated management for immunocompromised individuals. This finding refines risk stratification and clinical decision-making for vulnerable populations.

In their summary of MPXV research, Chen et al. discussed the recent advances in MPXV virology, pathogenesis, molecular evolution, clinical features, and diagnostic techniques. They also highlighted the public health challenges, including vaccine inequity, immunity gaps, surveillance limitations, vulnerabilities in the healthcare system, and stigma-related barriers to disease reporting and control.

We want to thank all the authors and reviewers for their valuable contributions to this Research Topic, and we hope that this collection of reviews and original articles will be helpful for clinicians and researchers.

